# Neutrophil Extracellular Traps: A Perspective of Neuroinflammation and Complement Activation in Alzheimer’s Disease

**DOI:** 10.3389/fmolb.2021.630869

**Published:** 2021-04-08

**Authors:** Gabriela Canalli Kretzschmar, Valéria Bumiller-Bini, Miguel Angelo Gasparetto Filho, Yohan Ricci Zonta, Kaio Shu Tsyr Yu, Ricardo Lehtonen R. de Souza, Luciane Alarcão Dias-Melicio, Angelica Beate Winter Boldt

**Affiliations:** ^1^ Laboratory of Human Molecular Genetics, Postgraduate Program in Genetics, Department of Genetics, Federal University of Paraná (UFPR), Belém, Brazil; ^2^ Medical School of Botucatu, Laboratory of Immunopathology and Infectious Agents–LIAI, UNIPEX–Experimental Research Unity, Sector 5, São Paulo State University (UNESP), Botucatu, Brazil; ^3^ Laboratory of Polymorphism and Linkage, Department of Genetics, Federal University of Paraná, Curitiba, Brazil; ^4^ Medical School of Botucatu, Department of Pathology, São Paulo State University (UNESP), Botucatu, Brazil

**Keywords:** Alzheimer's Disease, neutrophil extracellular traps, inflammation, complement system, CR1, C5a, C1q

## Abstract

Complement system (CS) components are associated with Alzheimer’s disease (AD), the commonest cause of dementia in the world. Neutrophils can be attracted to amyloid-β plaques by several pro-inflammatory factors, including the complement anaphylatoxin C5a. They may release neutrophil extracellular traps (NETs), which are chromatin nets associated with myeloperoxidase, elastase, and other enzymes. Some CS molecules, such as C5a, C1q, and CR1, are associated with increased neutrophil recruitment and NETs release. However, the relationship between CS molecules and NETs in AD is poorly understood. In this work, we detected higher NET concentrations in plasma and serum of Brazilian AD patients, than in elderly controls (medians = 2.78 [2.07–6.19] vs. 2.23 [0.33–4.14] ng/mL, *p* = 0.0005). We discussed these results within the context of our former findings on complement and AD and the context of the literature on complement and NET release, suggesting both as possible therapeutic targets to prevent the progress of the disease.

## Introduction

Neuroinflammation is a well-established phenomenon in AD (Czirr and Wyss-Coray, 2012; Wyss-Coray and Rogers, 2012; Dansokho and Heneka, 2018; Kloske and Wilcock, 2020) whose mechanisms are still poorly understood. They are related to the accumulation of amyloid-β (Aβ) plaques and neurofibrillary tangles (NFTs), characteristic AD biomarkers. The first present damage-associated molecular patterns (DAMPs) (Wyss-Coray and Rogers, 2012; Heppner et al., 2015) which are recognized by the complement system (CS) (McGeer et al., 1989; Veerhuis et al., 2011; Tenner et al., 2018). They also induce the expression of endothelial adhesion molecules and the release of pro-inflammatory cytokines by stimulated glial cells (reviewed in Pietronigro et al., 2017). Indeed, the CS appears to play a relevant role in AD, as judged by the strong association of complement genetic polymorphisms with this disease (Morgan, 2018; Krance et al., 2019; Kretzschmar et al., 2020; Tenner, 2020). Besides that, the CS has already been correlated with the formation of neutrophil extracellular traps (NETs) (Palmer et al., 2016; de Bont et al., 2019). NETs are composed of chromatin fibers, citrullinated histones, and cytoplasmic enzymes as myeloperoxidase (MPO) and neutrophil elastase (NE), which altogether operate as an extracellular platform for trapping and killing bacteria (Brinkmann et al., 2004; Yousefi et al., 2009). They have been observed in AD patients and an AD animal model (Zenaro et al., 2015; Dong et al., 2018). However, the possible role of the CS in NET release within AD has never been discussed before. In this study, we focused on reported interactions of the CS with Aβ plaques and their possible role in neutrophil recruitment and NETs release in AD. We also confirmed the presence of higher NET levels in an AD Brazilian cohort.

## Alzheimer’s Disease and the Complement System

AD is a neurodegenerative disease responsible for the largest number of dementia cases globally (Alzheimer’s Association, 2020). Several authors sought to understand the disease’s etiology by analyzing different pathways and metabolic processes, most of them causing or being influenced by neuroinflammation (Zlokovic, 2011; Heppner et al., 2015; Du et al., 2018). In AD, the production of Aβ plaques and NFTs are exacerbated. The accumulation of Aβ plaques in the extracellular environment causes the loss of interneural communication (synapses) and activates the local and systemic immunological responses since Aβ plaques can be recognized as DAMPs by phagocytic cells (Czirr and Wyss-Coray, 2012; Wyss-Coray and Rogers, 2012; Heppner et al., 2015) and activate the CS (McGeer et al., 2016).

The CS is considered a key element in innate immunity, playing an essential role in regulating and protecting the central nervous system. The CS consists of an enzymatic cascade with the participation of more than 50 circulating proteins, as well as soluble or membrane receptors and regulators (Lee et al., 2019). It can be activated by three different pathways: classical (CP), lectin (LP), and alternative (AP). For more information on activation and participating CS elements, consult the review of Ricklin et al., 2016. Although the complement pathways are activated in different ways, they all enhance phagocytic activity and may progress to the formation of membrane attack complexes (MAC), leading to cell lysis. In addition, the activation of the complement cascade also results in anaphylatoxin production and recruitment of inflammatory cells. The CS must be tightly regulated. If out of control, the cascade may become offensive, permanently injuring surrounding tissues (Ricklin et al., 2016).

**FIGURE 1:**
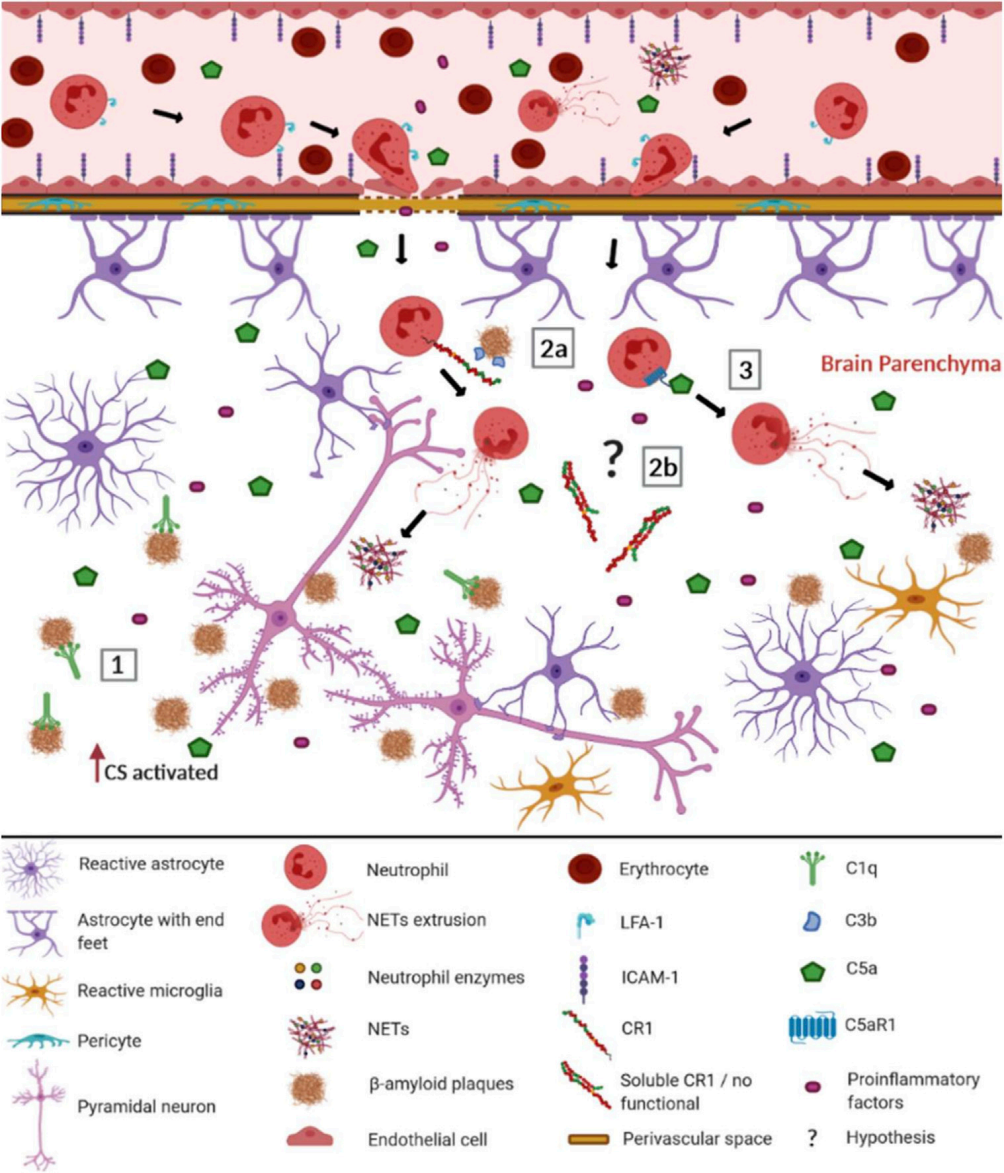
Possible involvement of the complement system (CS) in the recruitment of neutrophils and the formation of NETs in Alzheimer's disease. When the blood-brain barrier (BBB) is intact, most complement molecules do not pass through it but can be produced constitutively by brain cells. In AD, the BBB is compromised, facilitating the passage of complement elements, among other pro-inflammatory elements (Alexander, 2018). Aβ plaques are recognized as DAMPs by CS components and other innate immunity receptors, leading to the activation of BBB endothelial cells. They up-regulate ICAM-1 adhesion molecules, allowing neutrophils to adhere and interact with LFA-1, invading the cerebral parenchyma (Pietronigro et al., 2017). There the neutrophils worsen neuroinflammation, performing NET extrusion. In this context, the CS may be involved trough: (1) C1q binding to the Aβ plates, activating the classical complement pathway; (2a) CR1 (CR1*A) molecules present in the neutrophil membrane, that recognize the Aβ plates opsonized with C3b fragments, preferentially leading to NET extrusion; (2b) soluble CR1 formed by non-functional isoforms (CR1*B) that do not inhibit the CS (3) Potent anaphylatoxins such as C5a, which recruit neutrophils from the periphery to the Aβ plaque, being recognized by C5aR1 neutrophil receptors, promoting the release of NETs. Although other elements of the inflammatory reaction do occur, in this figure, we focus on elements of the CS discussed throughout this study. The complement cascades’ reactions were not represented due to space and number of elements participating in the pathways. The role of cytokines and endothelial adhesion molecules in neutrophil recruitment for Aβ plaques was extensively reviewed by Pietronigro et al. (2017) ICAM-1- Intercellular adhesion molecule 1; LFA-1- Lymphocyte function-associated antigen 1; NET- extracellular neutrophil traps; CS- complement system; C1q- complement component 1q; CR1- complement receptor 1; C3b- complement component 3b; C5a- complement component 5a; C5aR1- complement component 5a receptor. This figure was created with BioRender.com.

It is not a novelty that CS components and genetic polymorphisms are associated with AD (McGeer et al., 1989; Webster et al., 1997; Loeffler et al., 2008), as complement component 3 (Stoltzner et al., 2000; reviewed by Wyss-Coray and Rogers, 2012; Goetzl et al., 2018), complement component 4 (reviewed by Wyss-Coray and Rogers, 2012; Goetzl et al., 2018), complement membrane complex C5b-C9 (Goetzl et al., 2018), complement component 3a (C3a) and its receptor C3aR (Lian et al., 2015; Litvinchuk et al., 2018), complement component 5a (C5a) and its receptor C5aR1 (An et al., 2018), complement receptor 1 (CR1) (Lambert et al., 2009; Kretzschmar et al., 2020), clusterin (CLU) (Lambert et al., 2009) and complement component 1q (C1q) (Stoltzner et al., 2000; reviewed by; Wyss-Coray and Rogers, 2012; Lian et al., 2016; McGeer et al., 2016; Dejanovic et al., 2018; Goetzl et al., 2018), complement component 9 (reviewed by Wyss-Coray and Rogers, 2012), factor B and factor D (Goetzl et al., 2018). In this work, we will focus only on the CS components associated with AD, which may be related to the recruitment of neutrophils and the formation of NETs (Figure 1).

## Alzheimer’s Disease and Neutrophil Traps

Neutrophils have an essential role in inflammation, acting through many mechanisms: phagocytosis, degranulation, and extrusion of NETs (reviewed by Shen et al., 2001; Rossi et al., 2020). NETs are involved in host tissue injury and inflammation associated with autoimmune diseases, acute injuries, atherosclerosis, vasculitis, and cancer (Jorch and Kubes, 2017). In 2015, Zenaro and colleagues observed NETs adjacent to Aβ plaque deposits in the cerebral vascular and intraparenchymal region of an AD animal model and European AD patients. They suggested that Aβ plaques may play an essential role in the recruitment and movement of neutrophils, which Baik et al. (2014) also observed in an AD animal model. Furthermore, NET extrusion was also detected in high concentrations in European AD patients’ serum (Dong et al., 2018).

### C5a-C5aR1 Axis and the Relation With Neutrophil Traps and Alzheimer’s Disease

After complement activation (McGeer et al., 1989; Veerhuis et al., 2011), C3 and C5 are cleaved, generating the anaphylatoxins C3a and C5a (reviewed by de Bont et al., 2019). C3a lacks chemotactic activity (Ehrengruber et al., 1994), but C5a generates a potent chemotactic response and induces neutrophil migration (Ehrengruber et al., 1994), as well as the release of NETs (reviewed by de Bont et al., 2019). Therapeutic inhibitors blocking C5a and/ or its receptor C5aR1 have been proposed to treat AD (Fonseca et al., 2013; Landlinger et al., 2015; An et al., 2018). Although the functional connection between high NET levels and C5a has been well described (Yousefi et al., 2009; Huang et al., 2015; Yuen et al., 2016; Fattahi et al., 2018; de Bont et al., 2019), it has never been explored in AD.

The CS seems to be, at least through C5a, attracting neutrophils to the brain and inducing NETs extrusion. Neutrophil activation by C5a extrudes the mitochondrial DNA (Yousefi et al., 2009), which suggests that in AD, after NETs extrusion, the neutrophils do not die, at least not through C5a stimulation. NETs deposition triggers CS activation via the alternative pathway and properdin binding (Yuen et al., 2016). The neutrophils probably recognize the Aβ plaques and trap them, resulting in increased inflammation and tissue injury. After triggering the neutrophils to the tissue, the CS may not degrade the NETs, resulting in tissue NET accumulation and CS over-activation (de Bont et al., 2019). Thus, the use of C5a inhibitors may decrease NETs activation in AD.

### CR1 and Its Relationship With Neutrophil Traps and Alzheimer’s Disease

Even before NET’s discovery, several authors sought to understand the mechanisms of interaction between the complement system and neutrophils. Cytokines as tumor necrosis factor-alpha (TNFα), granulocyte-monocyte and granulocyte colony stimulating factors (GM-CSF, G-CSF), interleukin 1(IL-1), platelet activating factor (PAF), and lymphotoxin-beta (LTB) up-regulate phagocytic complement 1 receptor (CR1, also known as CD35) in neutrophils, increasing its association with C3b-opsonized microspheres. However, only TNFα, G-CSF, and PAF increased their phagocytic uptake (Ogle et al., 1990). Besides that, CR1 recognizing C3b-IgG complexes on the neutrophil membrane led to changes in signal transduction events associated with Fc receptors, resulting in myeloperoxidase's release and generation of hypochlorous acid (Sambandam and Chatham, 1998). CR1 blockage is followed by a decrease in NET concentration, revealing an essential role of this molecule in the extrusion process (Palmer et al., 2016). Thus, recognition of C3b-opsonized particles by CR1 on neutrophil membranes may preferentially lead to the release of NETs instead of phagocytosis, although more studies are needed to corroborate this hypothesis.

Interestingly, increased AD susceptibility has been repeatedly associated with *CR1* polymorphisms (Lambert et al., 2009). Nowadays, the hypothesis proposed for this association is that some polymorphisms (such as rs6656401*A) facilitate non-homologous recombination resulting in the preferential expression of dysfunctional CR1*B isoform (Brouwers et al., 2012; Mahmoudi et al., 2015). Although CR1*B has an additional C3b/C4b binding site, its neuronal expression occurs in vesicular form (Hazrati et al., 2012). It is also expressed in lower amounts in erythrocytes than the CR1*A functional protein (Mahmoudi et al., 2018). Thus, CR1*B probably impairs the process of removing Aβ plates and regulating the CS (Mahmoudi et al., 2018). Heterozygote CR1*A/CR1*B individuals express both isoforms. In this case, neutrophil recognition of C3b-opsonized Aβ plates may occur by CR1*A, with consequent release of NETs. CR1 can also be found in a soluble form (sCR1). AD patients have higher levels of sCR1 in serum (Mahmoudi et al., 2018), plasma (Kretzschmar et al., 2020), and cerebrospinal fluid (CFS) (Daborg et al., 2012). sCR1 is a potent local inhibitor of the complement system and is formed through vesiculation or proteolysis of the membrane-bound CR1 (Pascual et al., 1993; Danielsson et al., 1994; Dervillez et al., 1997; Hamer et al., 1998), inhibiting the CS by dissociating C3 convertases, and targeting C3b and C4b for degradation (Zhu et al., 2015). It is possible that large sCR1 quantities would inhibit complement’s beneficial role of removing Aβ plaques, contributing to its accumulation. However, no studies to date demonstrated whether individuals who have only the CR1*B isoform present functional sCR1. If sCR1 is generated from CR1*B, CS inhibition probably will not occur properly, recruiting more neutrophils to the affected region, with higher extrusion of NETs, ultimately contributing to chronic neuroinflammation. Although CR1’s participation in the increase of NETs release seems to be evident, it is not yet clear how this may be related to the different isoforms of the molecule and its association with AD. Still, it raises an exciting possibility of a new role for CR1 in the disease, which needs to be investigated in further functional studies.

### C1q and the Relation With Neutrophil Traps and Alzheimer’s Disease

The C1q molecule of the CP participates within an essential process in brain homeostasis. In periods when synapse pruning happens, C1q tags inappropriate connections between neurons for removal by the microglia (Presumey et al., 2017). In neurodegenerative diseases, C1q may lead to aberrant synapse loss (Dejanovic et al., 2018). Curiously, Aβ binds and activates C1q in the absence of immunoglobulins (Rogers et al., 1992), starting the CP and probably promoting synapse loss. A study with a mouse model of AD lacking C1q demonstrated a significant reduction in inflammation and neuropathological features (Presumey et al., 2017).

Some researchers already analyzed the relationship between C1q and NETs. Increased C1q deposition inhibits DNase activity, resulting in NET accumulation (Leffler et al., 2012). When C1q is inhibited, the complement cascade does not progress, and NETs do not appear (Hair et al., 2018). NETs are mainly degraded by endonuclease DNase1 (Hakkim et al., 2010) and then cleared by macrophages (Farrera and Fadeel, 2013). DNases have already been used to successfully treat AD in a case report (Tetz and Tetz, 2016). Genetic *DNASE1* variants have been investigated in systemic lupus erythematosus (Pruchniak et al., 2019), however, its role in AD has never been investigated. DNase has been used as an efficient drug to degrade NET structure in breast cancer, lung injury, and lupus mouse models (reviewed in Jorch and Kubes, 2017).

### High Neutrophil Traps Levels in a Brazilian Cohort

Recently, we confirmed the genetic association of complement receptor 1 (*CR1*) polymorphisms in an AD Brazilian cohort (Kretzschmar et al., 2020). Based on the association between molecules of the complement system and NETs, we aimed to investigate if NET levels are also increased within the same Brazilian cohort. We quantified NETs in plasma of 22 AD patients and 20 elderly controls (EC), and serum of another 11 EC (considering that NET levels are similar in serum and plasma of the same individual (Abrams et al., 2019)). The study was approved by the local ethics committee (CAAE 55965316.1.0000.0102). All participating individuals were older than 65 years (AD median = 82.5 [70–88] years old; EC median = 76 [69–99] years old). AD were recruited from the Clinical Hospital of the Federal University of Paraná. AD and EC were diagnosed or confirmed to be neurologically normal based on clinical history and cognitive tests (Frota et al., 2011). Diabetes and systemic arterial hypertension (SAH) are common pathologies in the elderly that may cause NET release (Soongsathitanon et al., 2019; Parackova et al., 2020). Both diseases were not associated with NETs in our study and the association of Alzheimer's with NETs was also independent of HAS (OR = [95%CI = 1.75–6.91], *p* = 0.003) (data available in Supplementary Table S1). The NE-DNA concentrations in serum and plasma samples were quantified using an adapted ELISA test with immunofluorescence (Czaikoski et al., 2016; Colón et al., 2019) (Supplementary Figure S1). Anti-elastase antibodies were used for capturing these NE-DNA complexes, and dsDNA fluorescent reagent was used for detection and quantification. The data was tested for normality (D’Agostino and Pearson test). We compared patients and controls in two ways: 1) using the absolute values (ng/mL), and 2) establishing the 3rd quartile in controls as a threshold for defining high and low NET levels (2.548 ng/mL). We also used this threshold for defining the theoretical median in the Wilcoxon test. The groups were compared using unpaired T-test and two-way ANOVA. All the analyses were done using GraphPad Prism v.6 software. The *p*-values were corrected for multiple testing using the false discovery rate (FDR) method (Benjamini and Hochberg, 1995), performed in R language 3.6.1, through the Stats package (R Development Core Team, 2011). Corrected *p*-values lower than 0.05 were considered significant. The data used is available in the Supplementary Table S1.

**FIGURE 2:**
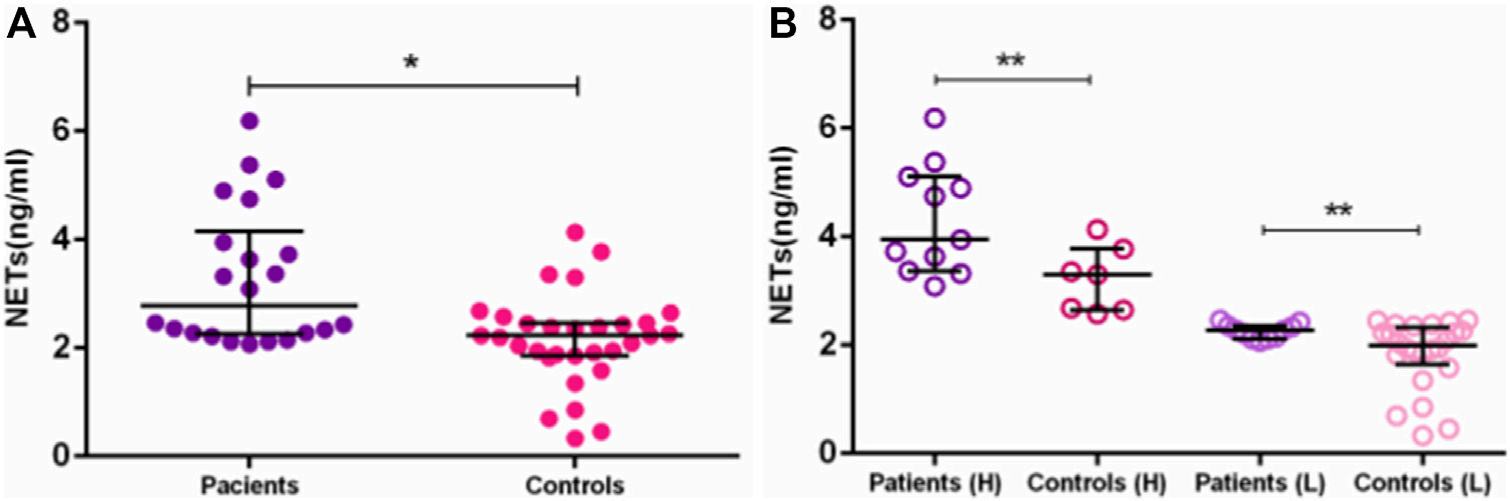
NETs concentration between AD patients and controls. Differences in NETs concentration between: **(A)**—AD patients and elderly controls. **(B)**—AD patients and elderly controls in High (H) and Low (L) groups. *p ≤ 0.01; **p ≤ 0.05.

Our study brings, for the first time, higher circulating NET levels in AD Brazilian patients. In this work, we detected higher NET concentrations in plasma and serum of Brazilian AD patients, than in elderly controls (medians = 2.78 [2.07–6.19] vs. 2.23 [0.33–4.14] ng/mL, *p* = 0.0005) (Figure 2A). The difference remained after dichotomizing AD and EC into high- and low-NET producers. The median NET concentration of high-NET producers was 3.95 (3.09–6.19 ng/mL) in AD, compared to 3.0 (2.586–4.14 ng/mL) in EC (*p* = 0.012). For low-NET producers, the median level of AD was 2.28 (2.07–2.46 ng/mL), compared to 2.0 in EC (0.33–2.46 ng/mL) (*p* = 0.042) (Figure 2B). Although NETs extrusion can lead to cell death by NETosis, we did not perform the assays to evaluate it. Despite the small number of samples used here, few studies investigating NETs in AD patients were published. All of them confirm the increased NETs in AD (Zenaro et al., 2015; Dong et al., 2018).

## Concluding Remarks

NETs seem to be promising as new therapeutic targets for AD treatment. We propose more investigations into the connection between C5a, C1q, and CR1 with NETs in AD, as well as genetic associations studies to investigate variants in DNase genes (*DNASE1*, *DNASE2,* and *DNASE1L3*) that can result in a down-regulation of DNase expression in AD.

## Data Availability

The original data presented in the study are included in the article/Supplementary Material, further inquiries can be directed to the corresponding author.
