# Supplementary material for: Neutrophil Extracellular Traps: A Perspective of Neuroinflammation and Complement Activation in Alzheimer’s Disease
Source: Front Mol Biosci. 2021 Apr 8;8:630869. doi: 10.3389/fmolb.2021.630869 (PMC8060499; doi:10.3389/fmolb.2021.630869)
Supplement: Supplementary file 1 [file datasheet1.docx]

Supplementary Material

# Supplementary Table 1 –Distribution of NETs and comorbidities (Diabetes and SAH) between groups.

| **Group** | **NETs(ng/mL)** | **Diabetes** | **SAH** |
| --- | --- | --- | --- |
| **AD patients** | 2.354 | X | X |
|  | 3.637 |  |  |
|  | 4.905 | X |  |
|  | 4.748 | X | X |
|  | 2.464 | X |  |
|  | 6.188 |  | X |
|  | 2.073 |  | X |
|  | 2.120 | X |  |
|  | 3.325 | X |  |
|  | 3.731 |  |  |
|  | 3.372 |  |  |
|  | 2.339 | X |  |
|  | 2.276 |  |  |
|  | 3.090 |  | X |
|  | 5.108 |  | X |
|  | 5.374 | X | X |
|  | 2.151 |  | X |
|  | 2.276 | X |  |
|  | 2.214 |  | X |
|  | 2.433 |  |  |
|  | 3.950 |  | X |
|  | 2.120 |  | X |
| **Elderly Controls** | 1.822 |  |  |
|  | 2.464 | X | X |
|  | 2.042 | X | X |
|  | 1.948 | X | X |
|  | 2.261 | X | X |
|  | 2.198 | X | X |
|  | 1.885 |  | X |
|  | 2.229 |  | X |
|  | 2.088 |  | X |
|  | 2.354 |  | X |
|  | 2.386 | X |  |
|  | 2.448 | X | X |
|  | 3.778 |  | X |
|  | 2.386 | NA | NA |
|  | 2.652 | X | X |
|  | 4.138 |  | X |
|  | 2.433 |  |  |
|  | 1.916 | X | X |
|  | 1.854 |  | X |
|  | 3.356 | X | X |
|  | 3.304 | X |  |
|  | 2.576 |  | X |
|  | 0.329 |  |  |
|  | 0.456 |  | X |
|  | 0.857 | X | X |
|  | 0.697 | NA | NA |
|  | 2.231 | X | X |
|  | 1.943 | NA | NA |
|  | 1.350 |  |  |
|  | 2.685 |  | X |
|  | 1.582 |  |  |

# SAH = Systemic Arterial Hypertension; X = present; NA = not available.

# Supplementary Figure 1 - Adapted ELISA test performed for quantification of NETs in serum and plasma samples.


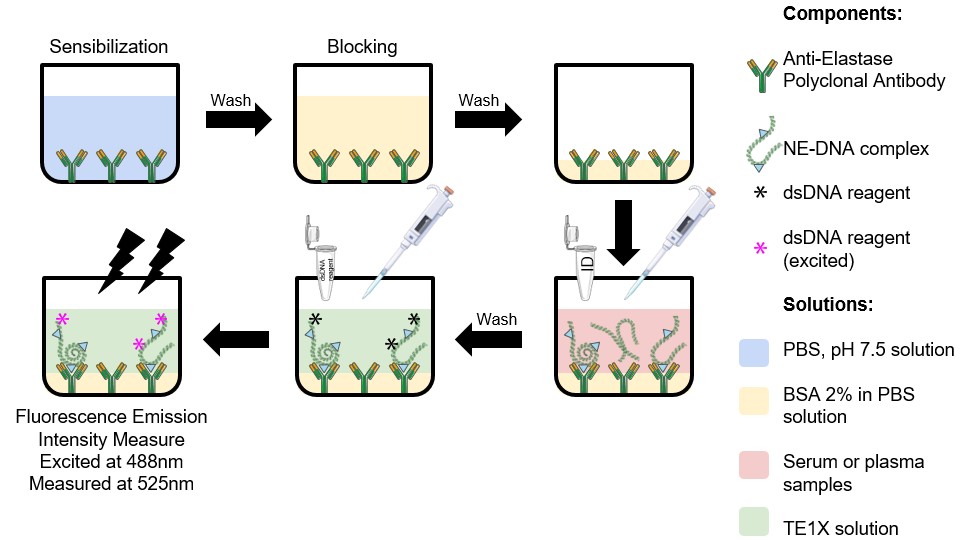


Bottom-transparent, 96-well plates were coated for 20 hours with anti-Elastase polyclonal antibody (Calbiochem, Cat. No. 481001). The plates were washed using detergent Tween-20 in PBS solution and blocked with bovine serum albumin (BSA) 2% in PBS solution during 2 hours at room temperature. After another washing session, the serum or plasma samples were added and incubated for 20 hours. The NE-DNA complexes are captured at their NE-end by anti-Elastase antibodies. Thus, the DNA-end of these complexes was used for detection. The plates were washed and the dsDNA reagent in TEx1 solution (Component A of the Quant-iT™PicoGreen dsDNA Reagent, Invitrogen) was added to the wells. The plates were incubated for 5 minutes in a dark environment before measurement. Using a microplate-reader Synergy™ H (BioTek), the samples were excited at 488nm and fluorescence emission intensity was measured at 525nm. A DNA standard curve was prepared in 8 wells of each plate using Lambda DNA standard (Component C of the Quant-iT™PicoGreen dsDNA Reagent, Invitrogen), with DNA concentrations ranging from 1000ng/ml to 7.8125ng/ml. The reading of the fluorescence emission intensity was interpolated within the DNA standard curve.
